# Clinical Profile and Outcome of Hospitalized Confirmed Cases of Omicron Variant of SARS-CoV-2 Among Children in Pune, India

**DOI:** 10.7759/cureus.24629

**Published:** 2022-04-30

**Authors:** Aarti A Kinikar, Sagar Vartak, Rahul Dawre, Chhaya Valvi, Pragathi Kamath, Naresh Sonkawade, Sameer Pawar, Vaishnavi Bhagat, Kiruthiga A, Komal Nawale, Isha Deshmukh, Rashmita Das, Rajesh K Kulkarni, Varsha Potdar, Rajesh Karyakarte

**Affiliations:** 1 Pediatrics, Byramjee Jeejeebhoy (BJ) Government Medical College & Sassoon General Hospitals, Pune, IND; 2 Microbiology, Byramjee Jeejeebhoy (BJ) Government Medical College & Sassoon General Hospitals, Pune, IND; 3 Pediatrics, Postgraduate Institute, Yashwantrao Chavan Memorial (YCM) Hospital, Pune, IND; 4 Infectious Disease, Indian Council of Medical Research (ICMR) - National Institute of Virology, Pune, IND

**Keywords:** clinical profile, outcomes, children, omicron variant sars- cov-2, hospitalized

## Abstract

Background

The Omicron variant of SARS-CoV-2 infection was seen to be more infectious but less severe in children than adults with reduced hospitalization rates. There is a paucity of data on hospitalized children with confirmed Omicron variant.

Objective

We describe demographic, epidemiologic, clinical, radiological, laboratory features and outcomes of children with confirmed Omicron variant of SARS-CoV-2 infection admitted to a tertiary care teaching hospital in Pune, India.

Methodology

Children who tested positive for SARS-CoV-2 - Omicron variant and were admitted between 1st December 2021 and 28th February 2022 were included in the study.

Results

Out of a total of 37 Covid-positive children admitted during the study period, 16 underwent genome sequencing of which 14 were confirmed to be Omicron variant and two were Delta variant. The age range was one month to 12 years and seven (50%) were male. Common presenting features were fever (n=13, 93%), cough (n=7, 50%), seizures (n=7, 50%) and coryza (n=5, 36%). Comorbidities noted were epilepsy (n=3, 21%) and one each with Thalassemia Major, suspected inborn error of metabolism (IEM), operated anorectal malformation with hypospadias, chronic suppurative otitis media with complications (mastoiditis and facial nerve palsy), neonatal cholestasis and intracranial bleed with dural venous sinus thrombosis. Malnutrition was noted in 42%, pallor in 10 cases (71%). Severe anaemia (n=10, 71%), elevated ferritin (n=6, 43%), positive C-Reactive Protein (n=4, 28%) and deranged D-dimer (n=11, 78%) were noted. The Neutrophil to Lymphocyte ratio (NLR) was >3.3 in five (36%) children. Four (28%) had evidence of pneumonia on the chest radiograph. Oxygen therapy was needed in nine (64%) while two children (14%) required mechanical ventilation. There were two deaths (14%) in children with multiorgan dysfunction and refractory shock. Intravenous immunoglobulin and methylprednisolone were administered to one patient respectively (14%). The median hospital stay was 10 days (Interquartile range = 8).

Conclusion

Hospitalized children with Omicron variant of SARS-CoV-2 who have underlying comorbidities may have severe presentations needing ICU care. Mortality rates are low with appropriate ICU care.

## Introduction

The severe acute respiratory syndrome coronavirus 2 (SARS-CoV-2), also known as the Coronavirus disease 2019 (COVID-19), pandemic in India has been characterised by three distinct waves (12th April 2020 to 14th January 2021, 10th March 2021 to 12th July 2021 and 28th December 2021 to 16th February 2022). In November 2021, a new SARS-CoV-2 variant was detected in South Africa which was associated with a rapid re-emergence of infections in Gauteng Province, South Africa. The World Health Organization (WHO) designated it as a variant of concern (Omicron variant) which eventually spread to 87 countries within a span of three weeks [1]. The Omicron variant was found to carry over 30 mutations in the spike glycoprotein, predicted to affect antibody neutralization and spike function [2]. In India, the peak cases in the second wave exceeded those in the first wave with a much steeper growth curve than the first wave. The third wave began on 28 December 2021 almost six months after the onset of the second wave and was steeper than the second wave, similar to the outbreaks driven by Omicron in several other countries [3]. Early studies suggest that infections by the Omicron variant might be of mild to moderate severity [4]. There is a lack of data on disease severity in children, although some published studies have observed that Omicron variant infection in children is comparatively less severe than the Delta variant [5,6]. Here we report the clinical profile and outcome of hospitalised, genome sequencing confirmed cases of the Omicron variant among children at a dedicated paediatric COVID-19 unit from the city of Pune, Maharashtra in western India.

## Materials and methods

Study site

The study was conducted at Sassoon General Hospital, a tertiary care hospital affiliated with B.J. Government Medical College, Pune, India.

Inclusion criteria

For this retrospective study, we identified hospitalized children between the age group of one month to 12 years who were genome sequenced confirmed cases of Omicron variant of SARS-CoV-2 infection between 1st December 2021 and 28th February 2022**.**

Exclusion criteria

Those children whose samples were not tested for genome sequencing were excluded from this study.

Data collection

This was a retrospective cohort study. This study was approved by the Institutional Ethics Committee of B.J. Government Medical College and Sassoon General Hospitals, Pune with an approval number BJGMC/IEC/Pharmac/ND-Dept 1020128-128 Date 01/03/2021. Data were obtained from medical case records. The following variables were collected: demographic information, including age, sex and geographic location, family clustering (one infected family member residing with the infant), COVID-19 vaccination profile of parents, presenting symptoms, duration of symptoms before presentation, comorbidities, the severity of the disease, laboratory parameters, chest radiograph findings, administered antiviral and antimicrobial therapies, duration of hospital stay and outcome.

Investigations done included hemogram, kidney/liver function test, C-reactive protein (CRP), procalcitonin, ferritin, D-dimer, lactate dehydrogenase (LDH), creatine kinase myocardial band (CK-MB), prothrombin time-international normalized ratio (PT-INR) and activated partial thromboplastin time (APTT).

Laboratory procedures

**Figure 1:**
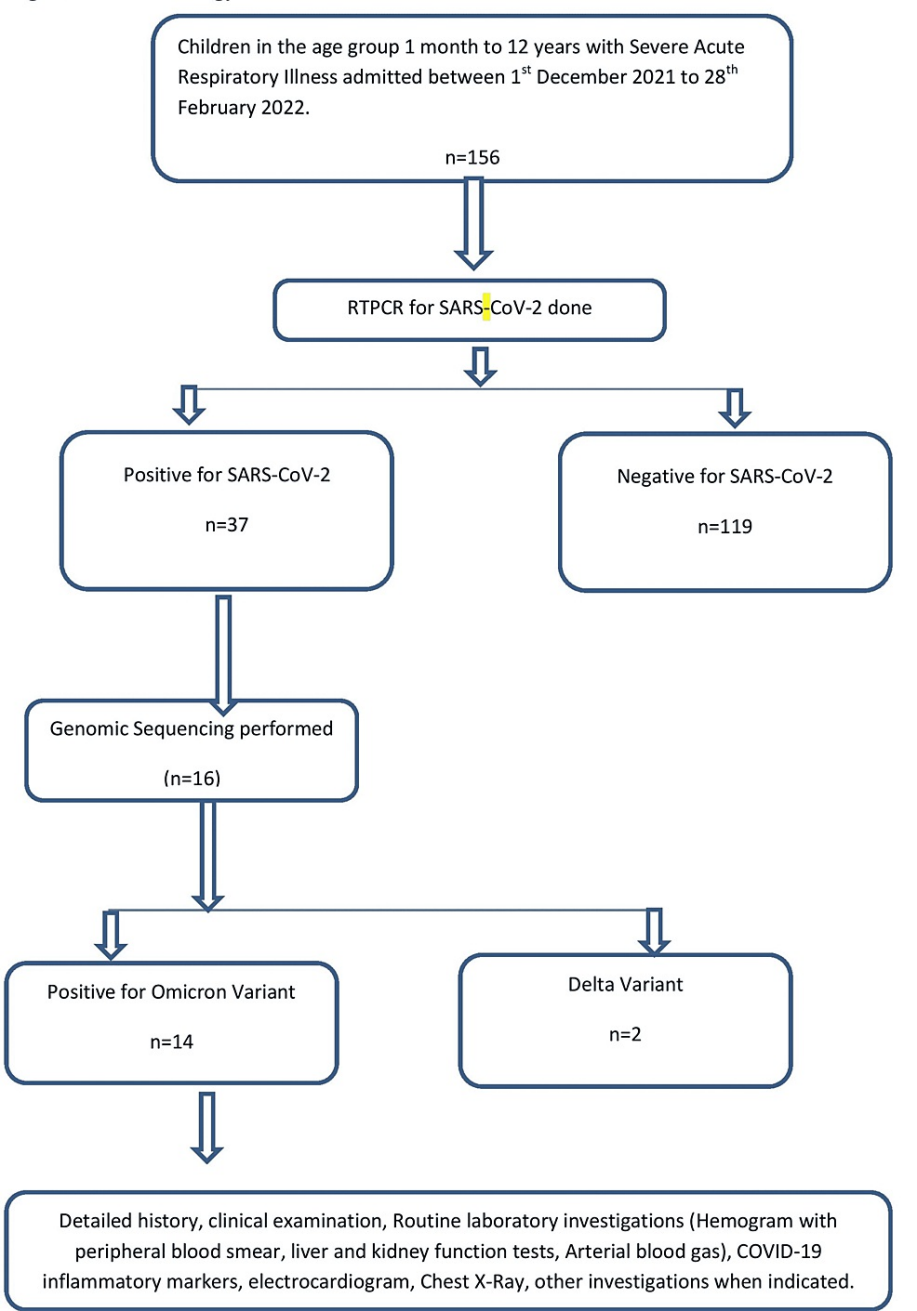
Study Flow Chart RT-PCR: Reverse Transcriptase-Polymerase Chain Reaction

Nasopharyngeal swabs were collected during hospitalization as per guidelines issued by the Government of India for testing suspected COVID cases. The genome sequencing was performed at the National Institute of Virology (NIV), Pune. The paediatric samples were tested for SARS-CoV-2 RT-PCR. The whole genomes were obtained using Oxford Nanopore Minion platform and Ion Torrent S5 platform using previously published protocol [7]. The MagMAX^TM^ Viral Nucleic Acid Isolation Kit was used to isolate RNA clinical samples (Thermo Fisher Scientific, Waltham, MA, USA). Library preparation and quantification was done as per the sequencing platform. The midnight expansion kit was employed for Oxford Nanopore. Sequencing data were processed using Commander S/w version 1.6.2 for Nanopore and Torrent Suite Software (TSS) v5.10.1 (Thermo Fisher Scientific, Waltham, MA, USA). Wuhan-HU-1 was used for reference-based genome assembly for all the samples. Figure 1 illustrates the study flow chart.

Statistical analysis

Statistical Package for the Social Sciences (SPSS version 20; IBM Inc., Armonk, NY, USA) software was used for data analysis. Categorical variables were presented as frequencies and percentages, whereas continuous variables were presented as median (Inter Quartile range, IQR).

## Results

We describe 14 confirmed, hospitalised cases of the Omicron variant of COVID-19 in the age group of one month to 12 years, at a dedicated paediatric COVID-19 unit in Pune, western India. Out of the 14 confirmed cases, seven (50%) were males. The cases were equally distributed under and over two years of age (n=7 under and over two years). All 14 were symptomatic and needed admission. Symptoms included fever (axillary temperature ≥ 37.5°C) in 13 (93%) children, cough in seven (50%), seizures in seven (50%) and coryza in five (36%). Other presenting symptoms were breathing difficulty, lethargy, vomiting, headache, jaundice, ear discharge and epistaxis in one case each.

Nine (64%) children had underlying chronic illness: three (21%) with epilepsy and one each with Thalassemia Major, suspected inborn error of metabolism (IEM), operated anorectal malformation with hypospadias, chronic suppurative otitis media with complications (mastoiditis and facial nerve palsy), neonatal cholestasis. One (7%) patient had an acute complication of intracranial bleed with dural venous sinus thrombosis. Varying grades of malnutrition (severe or moderate malnutrition) were observed in nearly 42% of children which could be attributed to the poor socioeconomic status and the underlying chronic illness.

Nine (64%) children required respiratory support on admission, of which seven needed continuous positive airway pressure (CPAP) while two required mechanical ventilation. The median duration of respiratory support in days was 1 (IQR=5).

Fathers of four (29%) children were completely vaccinated, eight (57%) were incompletely vaccinated and two (14%) were not vaccinated. Mothers of four (29%) children were completely vaccinated, four (29%) were incompletely vaccinated and six (42%) were not vaccinated.

Anaemia (Haemoglobin <11 g/dl) was observed in 10 (71%) children. The median haemoglobin level was 10 g/dl (IQR=3). The neutrophil to lymphocyte ratio (NLR) was >3.3 in five (36%) children. The median NLR was 1.6 (IQR=3.6).

COVID-19 inflammatory markers were done for all the patients which included C-reactive protein (CRP), lactate dehydrogenase (LDH), D-dimer, ferritin, CK-MB and interleukin-6 (IL-6). CRP of four (29%) children was positive. The median values of the above markers are illustrated in Table 1.

**Table 1:** COVID-19 inflammatory markers - normal range, median and interquartile range (IQR) LDH: Lactate Dehydrogenase, CK-MB: Creatine Kinase-Myocardial Band, IL-6: Interleukin-6

Parameter	Normal Range	Median	IQR
LDH (IU/L)	235-470	585	828
D-Dimer (mg/L)	0.1-0.5	2	2.11
Ferritin (ng/mL)	14-124	120	333.9
CK-MB (U/L)	0-24	33	46.9
IL-6 (pg/mL)	0.7	19	22.11

Total COVID-19 antibodies were raised in five (36%) children. It is known that antibody tests can become positive as early as one week after infection, the other possibility is that these children may have had COVID infection previously. X-ray findings were suggestive of bronchopneumonia in four (29%), hyperinflation in one (7%), while it was normal in nine (64%).

The median duration of hospital stay was 10 days (IQR=8). Out of the 14 children, there were two deaths (Case 4 and 12) while 12 children were discharged. Case 4 died on day 31 primarily due to sequelae of encephalitis and not due to Omicron, whereas Case 12 was a late referral for status epilepticus and succumbed within 24 hours of admission.

There were two deaths (Case 4 and 12) - the first was a four-month-old girl who presented with fever and seizures with encephalopathy, she required mechanical ventilation (at admission) for encephalopathy for 31 days and was diagnosed to have BA2 Omicron variant of SARS-CoV-2. The second case was a three-year-old known epileptic boy who had fever, coryza and presented to the hospital in status epilepticus which was refractory to treatment. Table 2 and Table 3 illustrate the clinical profile of the 14 children positive for the Omicron variant of COVID-19.

**Table 2:** Clinical profile of pediatric COVID-19 Omicron positive patients (Case 1-7) M: Male, F: Female, CRP: C-Reactive Protein, IVIG: Intravenous Immunoglobulin, Dexa: Dexamethasone, MPS: Methylprednisolone, CK-MB: Creatine Kinase Myocardial Band, CPAP: Continuous Positive Airway Pressure.

Parameters	Case 1	Case 2	Case 3	Case 4	Case 5	Case 6	Case 7
Age	4 months	18 months	6 years	4 months	9 years	10 years	6 years
Sex	M	M	F	F	F	M	M
Clinical presentation	Fever, noisy breathing	Fever, Seizures	Fever, Cough, Cold	Fever, Seizure	Fever, Headache, Vomiting	Fever, Seizure	Fever, Cough, Cold, Seizure
Parental vaccination status	Father - 1 dose, Mother - not vaccinated	Father - 2 doses, Mother - 2 doses	Father - 1 dose, Mother - 2 doses	Father - 2 doses, Mother - Not vaccinated	Father - 2 doses, Mother - 2 doses	Father - 1 dose, Mother - 1 dose	Father - 2 doses, Mother - 2 doses
Omicron Variant	BA.1	BA.1	BA.2	BA.2	BA.2	BA.2	BA.2
Covid markers							
CRP	Negative	Negative	Negative	Positive	Positive	Positive	Negative
CK-MB (U/L)	156	33	19	251	15	14	31.1
D-Dimer (mg/L)	0.5	3	10	11	3	2	0.8
Interleukin-6 (pg/mL)	9	19	33	4	92	35	14.14
Ferritin (ng/mL)	392	37	2000	1506	46	60	58
Lactate dehydrogenase (U/L)	2687	335	2880	2440	585	498	454
Co-morbidities	-	Anemia, Moderate acute malnutrition	-	Anemia	-	Epilepsy	Epilepsy, Klebsiella Pneumoniae sepsis, Anemia
Oxygen support	CPAP	No	CPAP	Mechanical ventilation	No	CPAP	No
X-Ray findings	Hyperinflation	Normal	Normal	Normal	Normal	Normal	Normal
IVIG/Dexa/MPS	-	-	-	IVIg 2 gms/kg	-	-	-
Hospital stay	25 days	6 days	5 days	31 days	6 days	6 days	14 days
Outcome	Discharged	Discharged	Discharged	Death	Discharged	Discharged	Discharged

**Table 3:** Clinical profile of pediatric COVID-19 Omicron positive patients (Case 8-14) M: Male, F: Female, CRP: C-Reactive Protein, IVIG: Intravenous Immunoglobulin, Dexa: Dexamethasone, MPS: Methylprednisolone, CPAP: Continuous Positive Airway Pressure, CK-MB: Creatine Kinase Myocardial Band.

Parameters	Case 8	Case 9	Case 10	Case 11	Case 12	Case 13	Case 14
Age	18 months	2 years	3 years	6 years	3 years	3 months	5 months
Sex	F	M	F	M	M	F	F
Clinical presentation	Cough, Cold, Lethargy	Fever, Cough, Cold	Fever, Cough	Fever, Headache, Ear Discharge	Fever, Cough, Cold, Seizures	Fever, Cough, Cold, Seizures	Fever, Jaundice, Epistaxis
Parental vaccination status	Father - Not vaccinated, Mother - Not vaccinated	Father - 1 dose, Mother - 1 dose	Father - 1 dose, Mother - Not vaccinated	Father - Not vaccinated, Mother - Not vaccinated	Father - 1 dose, Mother - Not vaccinated	Father - 1 dose, Mother - 1 dose	Father - 1 dose, Mother - 1 dose
Omicron Variant	BA.2	BA.2	BA.2	BA.2	BA.1	BA.2	BA.2
Covid markers							
CRP	Negative	Positive	Negative	Negative	-	Negative	Negative
CK-MB (U/L)	156	47.5	41	15	-	64.4	17.5
D-Dimer (mg/L)	1.24	1.2	20	0.89	-	2.42	0.17
Interleukin-6 (pg/mL)	76	18.85	5.2	12.89	-	111.9	20.6
Ferritin (ng/mL)	82	38.7	120	127	-	573	132
Lactate dehydrogenase (U/L)	461	1289	687	471	-	1187	442
Co-morbidities	Suspected inborn error of metabolism, anemia	Operated anorectal malformation, hypospadias, anemia, severe acute malnutrition	Chemical pneumonitis secondary to accidental paint thinner ingestion, anemia	Left-sided chronic suppurative otitis media, mastoiditis, severe wasting, anemia	Epilepsy, status epilepticus, anemia	Failure to thrive, anemia, intracranial bleed, dural venous sinus thrombosis	Failure to thrive, neonatal cholestasis, anemia, coagulopathy.
Oxygen support	CPAP	CPAP	No	No	Mechanical Ventilation	CPAP	CPAP
X-Ray findings	Diffuse bilateral infiltrates	Diffuse bilateral infiltrates	Diffuse bilateral Infiltrates	Normal	Normal	Diffuse bilateral infiltrates	Normal
IVIG/Dexa/MPS	-	-	-	-	MPS	-	-
Hospital stay	10 days	10 days	10 days	10 days	1 day	14 days	39 days
Outcome	Discharged	Discharged	Discharged	Discharged	Death	Discharged	Discharged

## Discussion

A new variant of SARS-CoV-2 was announced by the genomic surveillance team in South Africa on 24th November 2021. The variant was later given the name Omicron by the WHO [6]. With its doubling time of 1.2 days and genome sequencing, the Omicron variant revealed a higher number of non-synonymous mutations that included many mutations in the spike protein which have been proved to contribute to the transmissibility, severity of disease and immune system escape. More than 60 mutations have been identified which is the largest of all SARS-CoV-2 variants identified so far [8]. The spike mutations identified in Omicron are about 3-4 times more than those observed in the other Variants of Concern (VOC). It is worth noting that all the VOCs contain the amino acid change D614G in spike which, in previous studies, has been associated with increased upper respiratory tract viral loads and the younger age of patients [8]. The Omicron variant also has the mutation N501Y which is believed to enhance the attachment between the spike and angiotensin-converting enzyme 2 (ACE2) and higher transmissibility. In combination with the H69/V70 deletion, it might be increasing the transmissibility further [9].

In India, the third wave began on 28th December 2021, progressing rapidly and with a steeper curve than the previous waves [3]. Between 1st December 2021 to 28th February 2022, our dedicated paediatric COVID-19 unit had admitted 37 children in the age group of one month to 12 years who were COVID-19 RTPCR positive. The genomic sequencing of 14 children revealed Omicron Variant. Out of these 14 children, two (14%) required intensive care while others were admitted to the ward. The most common presenting symptoms were fever, cough and seizures. Seven (33%) children had generalised tonic-clonic seizures on presentation, of whom three had pre-existing epilepsy and one each was attributed to a typical febrile seizure, intracranial bleed with Dural venous sinus thrombosis and suspected IEM with hypoglycaemia. A study by Sharma et al. in Rajasthan, India showed most of Omicron patients (adult and paediatric) were asymptomatic (56.7%) or had mild disease (33%) while 9.2% had moderate symptoms and 0.7% had severe disease requiring hospitalization [10]. Two deaths in children under four years of age were reported from the US between December 19, 2021-January 31, 2022 when Omicron was the predominant variant. It was not clear if these cases were confirmed Omicron variant by genomic sequencing [11].

Oxygen therapy was required in nine (64%) children, two (14%) required invasive mechanical ventilation while seven (50%) required only CPAP. The median duration of oxygen therapy in days was 1 (IQR=5). Nine (64%) children had chronic illness: three (21%) with epilepsy and one each with Thalassemia Major, suspected inborn error of metabolism (IEM), operated anorectal malformation with hypospadias, chronic suppurative otitis media with complications (mastoiditis and facial nerve palsy), neonatal cholestasis. One (7%) patient had an acute complication: intracranial bleed with dural venous sinus thrombosis. Only one (7%) child was diagnosed with Multisystem Inflammatory Syndrome in Children (MIS-C) during hospitalisation with acute COVID and treated with IVIG. These co-morbidities were the reason for admission in these children, and might have affected the severity of COVID-19. Nearly 42% had associated varying grades of malnutrition.

The majority of the parents were incompletely COVID-19 vaccinated or not vaccinated at all, which can be hypothesized to contribute to the spread of the Omicron variant. Five (36%) children showed high total COVID-19 antibodies, which may indicate a reinfection by the Omicron variant, supporting the theory of immune escape.

The median duration of hospital stay was 10 days (IQR=8) which was shorter than that observed with previous variants. Of the 14 children, there were two deaths and 12 children were discharged, suggesting that the disease severity was mild to moderate in most of the children.

In this study, we found that the BA.2 variant was dominant (n=11, 79%). Other variants were BA.1 (n=3, 21%). Sharma et al. have demonstrated BA.1 (62.8%) was the dominant variant followed by BA.2 (23.7%) and B.1.529 (13.4%). From our study, it appears BA.2 to be the dominant variant suggesting changing variant dominance region-wise and as the wave progresses.

Our study is one of the few to describe the characteristics of genome sequencing confirmed cases of the Omicron variant among children. Our study has a limitation, that is, this is a descriptive case series without a comparator group. Larger studies of children with genome sequencing confirmed Omicron variants are needed.

## Conclusions

This study highlights that children, especially those with comorbidity, infected with Omicron variant are at a risk for hospitalization. A small proportion of these may need intensive care. With adequate monitoring and supportive care, the mortality rates are low (14 percent in our series).

The two deaths were with BA.1 and BA.2 variant in children with underlying comorbidity (anemia, epilepsy) and who presented to the hospital with severe symptoms. Early recognition and referral may have prevented mortality.
